# Drivers of tropical soil invertebrate community composition and richness across tropical secondary forests using DNA metasystematics

**DOI:** 10.1038/s41598-020-75452-4

**Published:** 2020-10-28

**Authors:** Katie M. McGee, Teresita M. Porter, Michael Wright, Mehrdad Hajibabaei

**Affiliations:** Department of Integrative Biology, Centre for Biodiversity Genomics, Biodiversity Institute of Ontario, University of Guelph, 50 Stone Road East, Guelph, ON N1G 2W1 Canada

**Keywords:** Biodiversity, Community ecology, Molecular ecology, Tropical ecology, Ecology, Computational biology and bioinformatics

## Abstract

Tropical forests are fundamental ecosystems, essential for providing terrestrial primary productivity, global nutrient cycling, and biodiversity. Despite their importance, tropical forests are currently threatened by deforestation and associated activities. Moreover, tropical regions are now mostly represented by secondary forest regrowth, with half of the remaining tropical forests as secondary forest. Soil invertebrates are an important component to the functioning and biodiversity of these soil ecosystems. However, it remains unclear how these past land-use activities and subsequent secondary forest developments have altered the soil invertebrate communities and any potential ecological consequences associated with this. DNA metabarcoding offers an effective approach to rapidly monitor soil invertebrate communities under different land-use practices and within secondary forests. In this study, we used DNA metabarcoding to detect community-based patterns of soil invertebrate composition across a primary forest, a 23-year-old secondary forest, and a 33-year-old secondary forest and the associated soil environmental drivers of the soil invertebrate community structure in the Maquenque National Wildlife Refuge of Costa Rica (MNWR). We also used a species contribution analysis (SIMPER) to determine which soil invertebrate groups may be an indication of these soils reaching a pre-disturbed state such as a primary forest. We found that the soil invertebrate community composition at class, order, family, and ESV level were mostly significantly different across that habitats. We also found that the primary forest had a greater richness of soil invertebrates compared to the 23-year-old and 33-year-old secondary forest. Moreover, a redundancy analysis indicated that soil moisture influenced soil invertebrate community structure and explained up to 22% of the total variation observed in the community composition across the habitats; whereas soil invertebrate richness was structured by soil microbial biomass carbon (C) (C_mic_) and explained up to 52% of the invertebrate richness across the primary and secondary forests. Lastly, the SIMPER analysis revealed that Naididae, Entomobryidae, and Elateridae could be important indicators of soil and forest recuperation in the MNWR. This study adds to the increasing evidence that soil invertebrates are intimately linked with the soil microbial biomass carbon (C_mic_) and that even after 33 years of natural regrowth of a forest, these land use activities can still have persisting effects on the overall composition and richness of the soil invertebrate communities.

## Introduction

Globally, forests are important ecosystems for hosting biodiversity and for providing ecosystem services for both the natural world (i.e. nutrient cycling) and for socioeconomic growth^1^. Despite their importance, forests worldwide are continually facing extraction-based land management practices such as the conversion to agriculture and infrastructure, which has contributed to a rise in greenhouse gas levels and species declines globally^2,3^. Human-mediated land conversion, such as deforestation and mining activities, not only results in changes in the plant communities, but also has serious consequences for soil abiotic and biotic properties, such that soils have a reduced capacity to sustain biological productivity which has implications for carbon sequestration and structural and functional biodiversity of soils^3–6^. Depending on the extent of disturbance in forests, some soils fail to recover and/or fall short of returning to their previous biotic status^7^. Tropical forests in particular are critical zones for global biogeochemical cycling and hotspots of biodiversity, despite these important ecosystems only constituting 7–10% of the Earth’s land surface^5,8,9^. The recuperation of soils in the tropics remains an important initiative for tropical forest recovery, as secondary forests serve the potential for soil carbon sequestration and refugia for biodiversity^10–13^. Thus, understanding the consequences these activities have had on the soil biotic ecological drivers in regenerating tropical forests warrants investigation.

The development of secondary forests in the tropics provides an avenue for the amelioration of degraded soils. However, previous land use histories are known to create persisting alterations to the plant communities, and also affect various components in the soil such as soil texture and soil nutrient content and availability^14^. These legacy effects often cause significant reductions of essential soil carbon (C) and nitrogen (N) through the loss of vegetation and through soil degradation. Through some of these secondary forest developments, it is believed that forest recovery, or afforestation, can help restore soil C and N; however, in the tropics it has been found that even landscapes of similar origin and past land use history can exhibit multiple successional pathways with differing routes of forest recovery^13,15^.

Soil microbial communities have been recently identified as key components to restoring ecological functions in degraded soils^16^. As with the soil microbial communities, the soil invertebrate communities are considered an important component of soil biodiversity and ecological function, and therefore are important to consider in the process of forest recovery following disturbance^17^. This is especially important when considering foodweb structure in soil and the position of invertebrates as an intermediate trophic layer between microbial taxa and higher-level animals. Despite their contribution towards soil biodiversity, extant studies regarding changes in the soil invertebrate community across regenerating secondary forests are substantially lacking^18^. Soil invertebrates play an essential role in the recycling of organic matter and are capable of enhancing decomposition and primary productivity for soil microbial communities, either directly or indirectly^19–21^. More specifically, soil invertebrate groups are capable of increasing soil microbial inoculation to the surrounding available soil nutrients through substrates rich in fecal material, and is typically a result of feeding methods and mobility characteristics^22^. For example, nitrogenous waste often in the form of ammonia (NH_3_), is released through fecal material of soil invertebrates such as collembolans or nematodes which is readily available to plants, bacteria and fungi^20,21,23–25^. Therefore, it is important to understand not only how the soil microbial communities change under these dynamics, but also how the soil invertebrate communities respond to land use change and emergence of secondary forest developments.

Soil invertebrates have complex relationships with the surrounding environment and are affected by variations in the microhabitat and fluctuations in the types and quality of resources^19–21,26^. For example, collembolan (springtail) richness is known to be negatively affected by soil disturbances, such that the number of collembolan individuals decreases as disturbance intensity increases^27^. Likewise, the soil environmental factors that can favor soil invertebrate richness are litter and soil quality (i.e. resource quality), soil C:N ratios, organic matter content, and soil microbial biomass^28^. Thus, soil invertebrates are good candidates for detecting and monitoring changes in terrestrial soil ecosystems. However, it is still unclear how soil environmental changes associated with changes in land management influence the soil invertebrate communities in tropical secondary forests. Identifying which soil abiotic factors most strongly drive soil invertebrate community richness it is therefore crucial to understand how to improve soil biomass development and secondary forest regeneration.

While DNA metabarcoding techniques have been widely applied to investigate soil microbial communities, the application of these techniques to survey bulk soil invertebrate communities in the tropics remains limited^29,30^. Traditional studies that use morphological strategies to identify invertebrate communities are typically based on samples collected from pitfall traps for ground dwelling arthropods and, Malaise traps for flying insects, with less focus on DNA metabarcoding from bulk soil samples^29,31,32^. Previous studies have used DNA metabarcoding to examine the diversity of specific invertebrate taxonomic groups such as collembolans, nematodes, and annelids; however, few studies have performed DNA metabarcoding of soil invertebrates for community-scale analyses and the associated soil abiotic drivers of the community^29,33–37^. Moreover, most work performed on soil analyses has been in temperate regions with limited focus on tropical areas^26,30^. With the advent of DNA metabarcoding, obtained sequences can be compared to a growing standard reference library of known organisms and the taxa present in an environmental sample such as soil can be identified with high confidence^38–42^. Here, we used DNA metabarcoding to analyze the soil invertebrate community composition from bulk-soil across a primary forest and two different ages of secondary forests in the Maquenque National Wildlife Refuge (MNWR) of Costa Rica.

In this study, we aimed to examine differences in the soil invertebrate composition at the community-scale (and at different taxonomic resolutions) and assess the associated soil abiotic drivers across a primary forest and two different ages of secondary forests. We asked three questions pertaining to the soil invertebrate community: (1) Are there differences in the soil CO1 community composition between a primary forest and two different secondary forests of different ages (at different taxonomic resolutions)? (2) Which soil taxa are contributing to the differences in the community composition? (3) Which soil abiotic factor(s) is (are) best explaining the differences in community composition? Here, we provide evidence that two different ages of secondary forest and a primary forest have relatively different soil invertebrate community compositions, and that even after 33 years of natural forest regrowth, secondary forest soils do not harbor as much soil invertebrate richness in comparison to the primary forest soils. Moreover, we provide evidence that soil microbial biomass C may be a strong determinant of soil invertebrate richness.

## Results

### Soil invertebrate community composition and richness

#### Class

**Figure 1:**
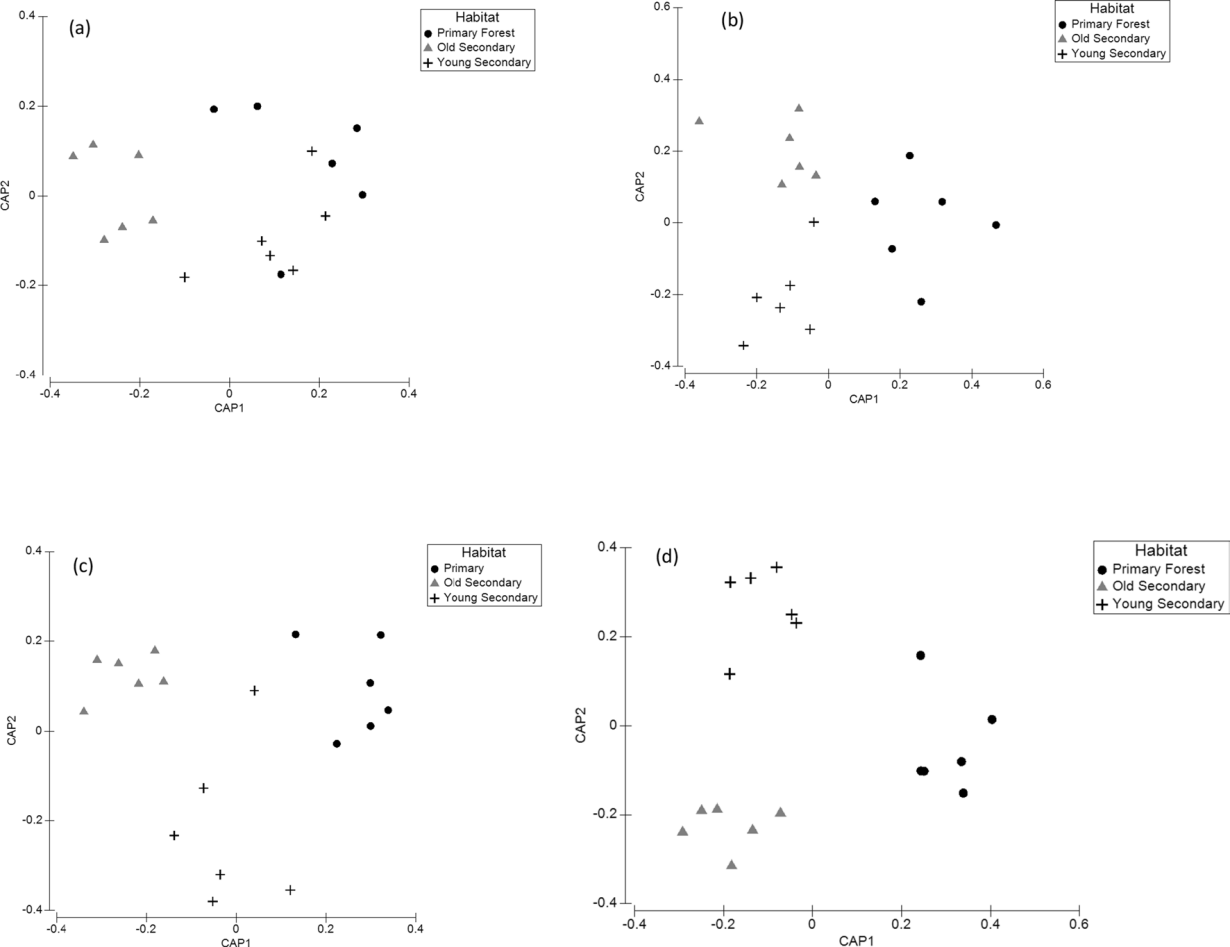
Canonical analysis of principle coordinates (CAP) ordination plot of the soil invertebrate (**a**) class, (**b**) order, (**c**) family, and (**d**) ESV level community composition across the primary forest, and two different ages of secondary forest in the MNWR.

**Figure 2:**
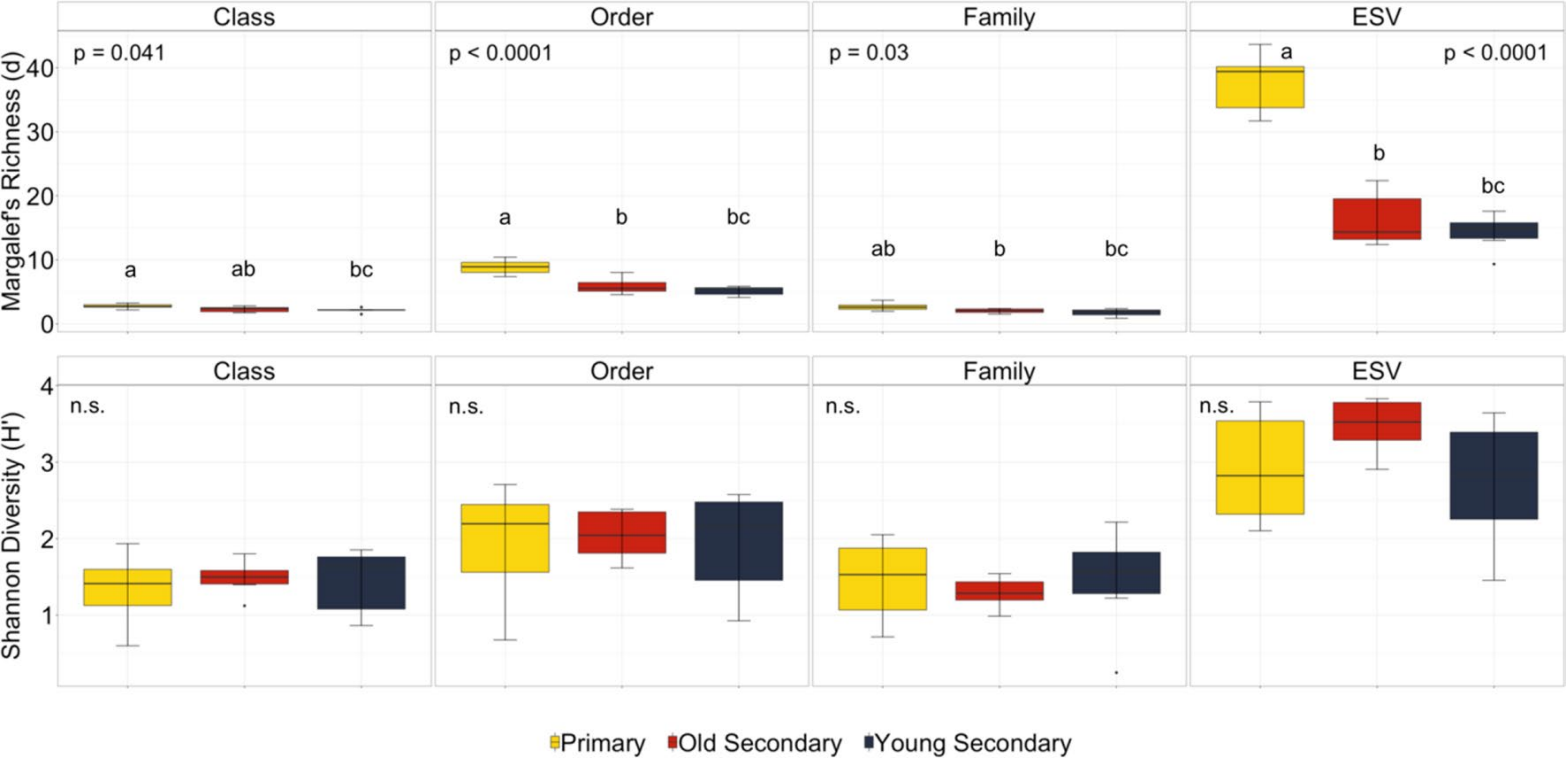
The one-way ANOVA results evaluating the mean values of Margalef’s richness and Shannon diversity of soil invertebrate class, order, family, and ESV resolutions across the primary forest, 33-year-old secondary forest (old), and the 23-year-old secondary forest (young) in the MNWR of Costa Rica. Different letters denote significant pairwise comparisons (*p* < 0.05) based on Tukey’s HSD post-hoc analyses.

The dominant CO1 classes included Clitellata and Polychaeta (Annelidae), Collembola, Insecta, Chilopoda, and Arachnida (Arthropoda), and Chromadorea (Nematoda) (Table S2). The PERMANOVA results indicated that the soil CO1 class community composition was mostly distinct across the habitats (Fig. 1a) (*p* < 0.05), except between the primary forest and the 23-year-old secondary forest (*p* > 0.05) (Table 1a). No differences were observed between habitats in the CO1 class diversity; however, CO1 class richness was significantly greatest in the primary forest in comparison to the 23-year-old secondary forest (F_2,15_ = 3.98, *p* = 0.041), but not between the primary and 33-year-old secondary, and not between the two secondary forests (*p* > 0.05) (Fig. 2).

**Table 1:** PERMANOVA of class, order, family, and ESV soil invertebrate community composition across habitats.

Habitat pairwise groups	Pseudo-F	*p* value	% Dissim	Cohen’s *d*
**(a) Class**				
Primary, old secondary	3.286	0.016	36.66	0.8545
Primary, young secondary	0.323	0.956	36.17	0.2678
Old secondary, young secondary	3.484	0.001	37.08	0.880
Primary, old secondary	2.952	0.007	51.21	0.8099
**(b) Order**				
Primary, young secondary	1.260	0.206	53.57	0.5292
Old secondary, young secondary	2.318	0.010	52.63	0.7177
Primary, old secondary	1.881	0.002	49.39	0.6465
**(c) Family**				
Primary, young secondary	1.106	0.293	54.52	0.4958
Old secondary, young secondary	1.880	0.005	50.59	0.6464
Primary, old secondary	2.260	0.004	88.56	0.7087
**(d) ESV**				
Primary, young secondary	1.335	0.003	87.86	0.5447
Old secondary, young secondary	1.798	0.004	87.00	0.6321

The SIMPER results indicated that the top 3 taxonomic classes responsible for the percent dissimilarity between the primary forest and old secondary were Clitellata (20%), Collembola (11.25%), and Insecta (10.93%). Differences between the old and young secondary were contributed by Clitellata (19.71%), Collembola (15.51%), and Arachnida (10.77%) (Table 2). The top 3 taxa responsible for species contribution between the primary and young secondary were Collembola (16.69%), Clitellata (13.96%), and Insecta (13.47%). An exhaustive list of the SIMPER taxonomic contributions can be found in the Supplementary Material (Table 2).

**Table 2:** The similarity of percentage analysis (SIMPER) using a one-way design with a 70% cut-off percentage to list only higher-contributing taxonomic groups for the soil COI class community composition between the three habitats in the MNWR (PF = primary forest, OS = 33-year-old secondary forest, YS = 23-year-old secondary forest).

Invertebrate class	Proportion of reads (%)		Average dissimilarity	Contribution (%)	Cumulative (%)
	Primary	Old secondary			
Clitellata	22.4988	48.9432	7.33	19.99	19.99
Collembola	19.1567	3.2123	4.12	11.25	31.23
Insecta	30.6732	24.9377	4.01	10.93	42.16
Chilopoda	0.8365	4.6633	3.33	9.09	51.25
Arachnida	10.8713	5.5153	3.01	8.21	59.47
Polychaeta	0.6159	5.0755	2.79	7.62	67.08
Chromadorea	9.8300	4.1655	2.72	7.41	74.50

Invertebrate class	Proportion of reads (%)		Contribution (%)	Cumulative (%)	Average dissimilarity
	Old secondary	Young secondary			
Clitellata	48.9432	14.5616	7.31	19.71	19.71
Collembola	3.2123	28.3412	5.75	15.51	35.22
Arachnida	5.5153	17.8634	3.99	10.77	45.99
Chilopoda	4.6633	0.5128	3.05	8.23	54.22
Insecta	24.9377	21.0466	2.89	7.79	62.01
Polychaeta	5.0755	5.9399	2.80	7.54	69.55
Malacostraca	1.2544	1.8981	1.86	5.03	74.58

Invertebrate class	Proportion of reads (%)		Average dissimilarity	Contribution (%)	Cumulative (%)
	Primary	Young secondary			
Collembola	19.1567	28.3412	6.04	16.69	16.69
Clitellata	22.4988	14.5616	5.05	13.96	30.65
Insecta	30.6732	21.0466	4.87	13.47	44.12
Arachnida	10.8713	17.8634	4.40	12.16	56.28
Polychaeta	0.6159	5.9399	2.97	8.21	64.50
Chromadorea	9.8300	4.1655	2.92	8.06	72.56

#### Order

The dominant CO1 orders included, Haplotaxida (Oligochaeta), Entomobryomorpha (Collembola), Coleoptera and Lepidoptera (Insecta) (Table S3). Soil invertebrate order community composition was significantly different between the primary forest and old secondary forest, and between the old and young secondary forest (*p* < 0.05) (Table 1b) (Fig. 1b). However, soil invertebrate order community composition was not different between the primary forest and young secondary forest (*p* = 0.21) (Table 1b). Soil invertebrate order diversity was not different in pairwise comparisons between habitats, but CO1 order richness varied significantly (F_2,15_ = 19.61, *p* < 0.0001) across habitats with the primary forest having significantly greater order richness in both the old and young secondary forest (*p* < 0.05) (Fig. 2). Order richness was not different between the old and young secondary forest (*p* > 0.05) (Fig. 2).

The SIMPER results indicated that the top 3 taxonomic orders responsible for the percent contribution between the primary forest and old secondary forest were Haplotaxida (9.31%), Entomobryomorpha (5%), and Coleoptera (4.15%). In the old and young secondary forest, Haplotaxida contributed to 9.81% of the dissimilarity, whereas Entomobryomorpha and Sarcoptiformes contributed to 7.67% and 5.74%, respectively (Table 3). Between the primary forest and young secondary forest, Entomobrymorpha (8.63%), Haplotaxida (6.58%), and Sarcoptiformes (5.48%) were the top 3 taxa that contributed the most to the percent dissimilarity between these two habitats (Table 3).

**Table 3:** The similarity of percentage analysis (SIMPER) using a one-way design with a 70% cut-off percentage to list only higher-contributing taxonomic groups for the soil COI order community composition between the three habitats in the MNWR (PF = primary forest, OS = 33-year-old secondary forest, YS = 23-year-old secondary forest).

Invertebrate order	Proportion of reads (%)		Average dissimilarity	Contribution (%)	Cumulative (%)
	Primary	Old secondary			
Haplotaxida	22.4470	48.8905	4.77	9.31	9.31
Entomobryomorpha	17.2021	3.0921	2.56	4.99	14.30
Coleoptera	11.3763	2.8190	2.13	4.15	18.45
Lepidoptera	0.8123	6.7572	2.00	3.91	22.36
Scolopendromorpha	0.0764	3.2119	1.78	3.47	25.83

Invertebrate order	Proportion of reads (%)		Average dissimilarity	Contribution (%)	Cumulative (%)
	Old secondary	Young secondary			
Haplotaxida	48.8905	14.2038	5.16	9.81	9.81
Entomobryomorpha	3.0921	26.4398	4.04	7.67	17.49
Sarcoptiformes	1.0886	11.3084	3.02	5.74	23.23
Lepidoptera	6.7572	3.3075	2.79	5.30	28.52
Phyllodocida	2.9642	4.2590	1.98	3.76	32.28

Invertebrate order	Proportion of reads (%)		Average dissimilarity	Contribution (%)	Cumulative (%)
	Primary	Young secondary			
Entomobryomorpha	17.2021	26.4398	4.63	8.63	8.63
Haplotaxida	22.4470	14.2038	3.53	6.58	15.22
Sarcoptiformes	0.6236	11.3084	2.94	5.48	20.70
Coleoptera	11.3763	1.7546	2.64	4.93	25.63
Blattodea	7.7094	2.2808	2.32	4.34	29.97

#### Family

Some of the dominant CO1 families included Enchytraeidea, Naididae, and Glossoscolecidae (Oligochaeta Haplotaxida), and Isotomidae (Collembola), (Table S4). The PERMANOVA results indicated that family community composition was significantly different between the primary forest and old secondary forest, and between the old and young secondary forest (*p* < 0.05) (Table 1c) (Fig. 1c). However, the family community composition was not different between the primary forest and young secondary forest (*p* > 0.05) (Table 1c). Family diversity across the habitats did not vary significantly between habitats (F_2,15_ = 0.19, *p* = 0.826), however, family richness was significantly greatest in the primary forest (2.68 ± 0.62) in comparison to the old and young secondary forests (F_2,15_ = 4.54, *p* = 0.03) (Fig. 2).

The SIMPER results indicated that Enchytraeidae (9.14%), Naididae (8.85%), and Elateridae (Coleoptera) (7.93%) were the CO1 families that contribute the most to the percent dissimilarity between the primary forest and old secondary forest (Table 4). Isotomidae (10.41%), Enchytraeidae (9.90%), and Onychiuridae (Collembola) (7.93%) contributed the most to the percent dissimilarity between the old and young secondary forest. Similarly, Naididae (10.30%), Isotomidae (9.40%), and Enchytraeidae (7.22%) contributed to the dissimilarity between the primary forest and young secondary forest (Table 4).

**Table 4:** The similarity of percentage analysis (SIMPER) using a one-way design with a 70% cut-off percentage to list only higher-contributing taxonomic groups for the soil COI family community composition between the three habitats in the MNWR (PF = primary forest, OS = 33-year-old secondary forest, YS = 23-year-old secondary forest)**.**

Invertebrate family	Proportion of reads (%)		Average dissimilarity	Contribution (%)	Cumulative (%)
	Primary	Old secondary			
Enchytraeidae	33.1271	61.7891	4.51	9.14	9.14
Naididae	19.3938	8.3140	4.37	8.85	17.99
Elateridae	12.3764	0.7656	3.91	7.93	25.92
Glossoscolecidae	2.5301	6.4460	3.59	7.27	33.19
Megascolecidae	8.6060	4.2472	3.57	7.23	40.42
Entomobryidae	7.8777	2.6743	2.93	5.93	46.35

Invertebrate family	Proportion of reads (%)		Average dissimilarity	Contribution (%)	Cumulative (%)
	Old secondary	Young secondary			
Isotomidae	0.5007	16.7991	5.27	10.41	10.41
Enchytraeidae	61.7891	36.2288	5.00	9.89	20.30
Onychiuridae	0.0000	6.8692	4.01	7.93	28.23
Naididae	8.3140	11.5921	3.80	7.52	35.74
Trhypochthoniidae	0.1897	6.2398	3.69	7.29	43.03
Formicidae	3.5395	4.3565	3.18	6.28	49.31

Invertebrate family	Proportion of reads (%)		Average dissimilarity	Contribution (%)	Cumulative (%)
	Primary	Young secondary			
Naididae	19.3938	11.5921	5.62	10.30	10.30
Isotomidae	0.7443	16.7991	5.12	9.40	19.70
Enchytraeidae	33.1271	36.2288	3.94	7.22	26.92
Trhypochthoniidae	2.0559	6.2398	3.82	7.01	33.93
Onychiuridae	2.1511	6.8692	3.68	6.75	40.68
Elateridae	12.3764	0.0000	3.60	6.60	47.28
Megascolecidae	8.6060	5.2660	3.51	6.44	53.72
Entomobryidae	7.8777	1.7974	3.43	6.30	60.02

### Exact sequence variants (ESVs)

The CO1 ESV community composition was significantly different between all pairwise habitat comparisons, indicating that ESV community composition is very distinct across the primary forest, old secondary forest, and young secondary forest (*p* < 0.05) (Table 1d) (Fig. 1d). The results of the one-way ANOVA indicated that the CO1 ESV richness was significantly greatest in the primary forest soils (37.75 ± 4.86) in comparison to the old and young secondary forest soils (F_2,15_ = 59.51, *p* < 0.0001) (Fig. 2). In contrast, the CO1 ESV diversity was not significantly different across habitat soils (F_2,15_ = 1.95, *p* = 0.18) (Fig. 2).

### Drivers of soil invertebrate community composition and richness

**Figure 3:**
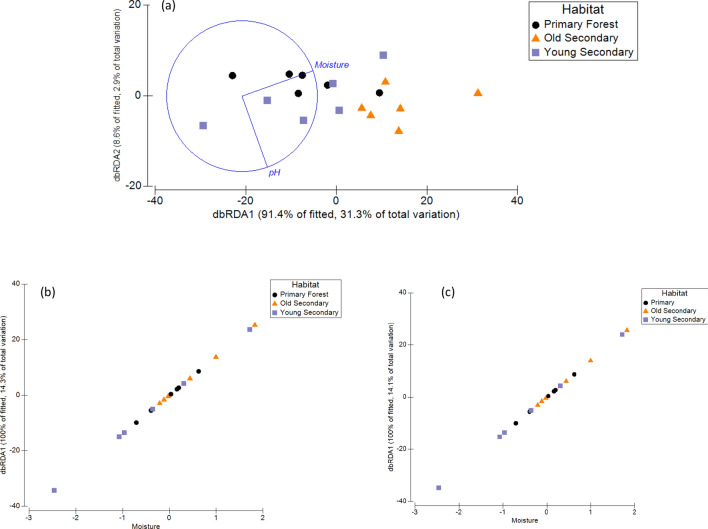
The distance-based redundancy analysis (dbRDA) for soil invertebrate (**a**) class, (**b**) order, and (**c**) family community composition. Soil moisture accounted for up to 22% of the total variation observed in the soil invertebrate community composition (*p* < 0.05; Table 5).

**Figure 4:**
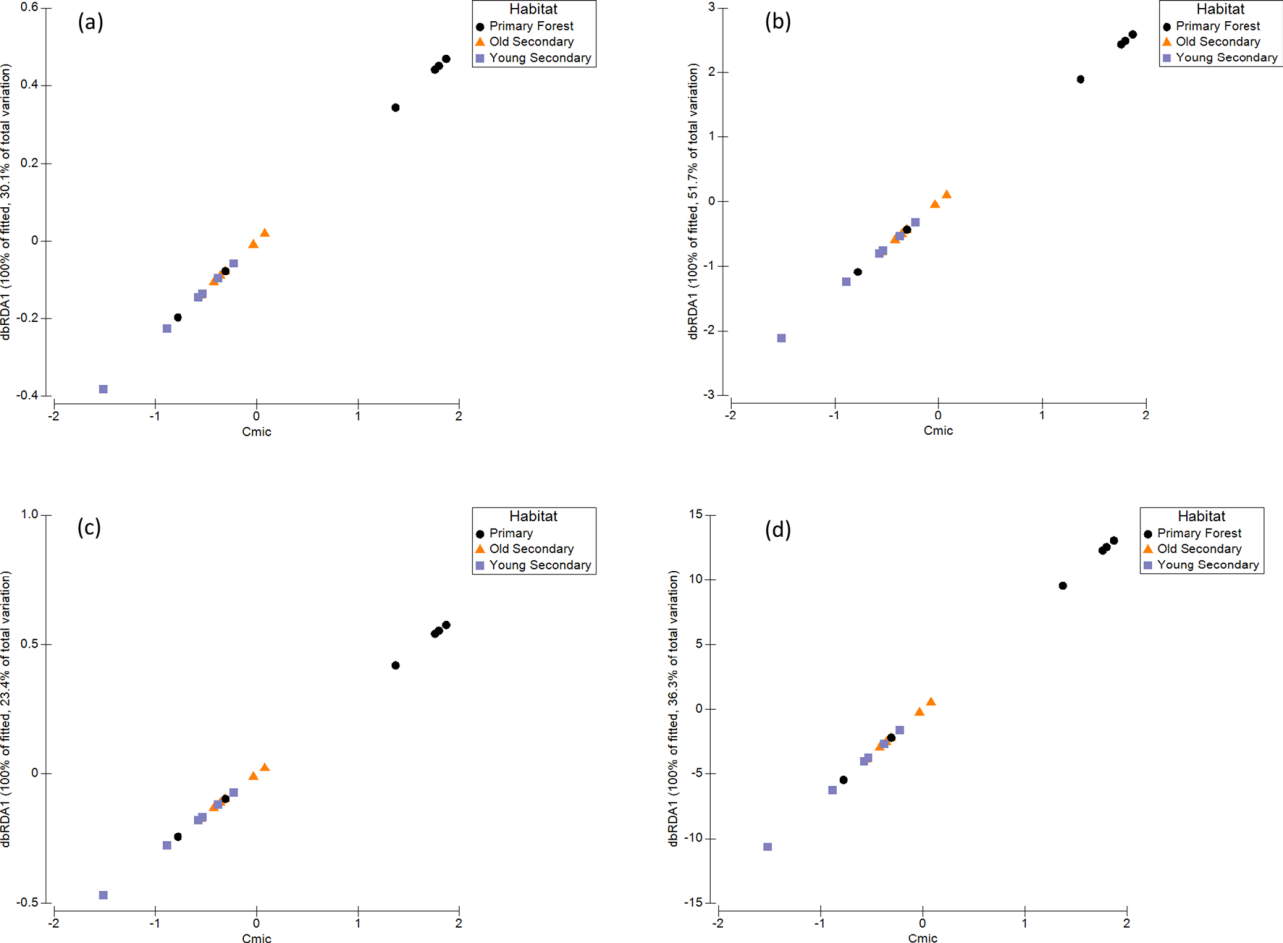
Distance-based redundancy ordination plot of the distance-based linear model sequential tests (DistLM) of the soil invertebrate (**a**) class, (**b**) order, (**c**) family, and (**d**) ESV richness to determine which soil abiotic variable is best explaining the patterns in the soil invertebrate richness across the primary forest and two different ages of secondary forest in the MNWR. Soil microbial biomass C accounted for up to 52% of the variation observed in the soil invertebrate richness (*p* < 0.05; Table 6).

Soil moisture and pH were the best variables explaining the invertebrate class community composition, and together, explained 34% of the total variation observed (Fig. 3a–c) (Pseudo-F = 4.5358, *p* = 0.002, AICc = 117.02 and Pseud-F = 2.7586, *p* = 0.012, AICc = 116.89, respectively) (Table 5a–d). Likewise, soil moisture also explained the invertebrate order and family community composition across the habitats, and explained 14.26% and 14.06% of the total variation observed (Fig. 3a–c), respectively (Pseudo-F = 2.660, *p* = 0.006, AICc = 130.39 and Pseudo-F = 2.618, *p* = 0.004, AICc = 131.51) (Table 5b,c). Soil moisture also explained the ESV community composition, however, the DistLM sequential tests could not verify a best solution, as including or excluding moisture did not improve the overall model (Table 5d). In contrast to the community composition, richness at the class, order, family, and ESV levels were best structured by soil C_mic_ and explained 30.06%, 51.73%, 23.41%, and 36.35%, respectively, of the total variation observed across the habitats (Table 6) (Fig. 4).

**Table 5:** A distance-based linear model (DistLM) of the marginal and sequential tests of the relationship between the soil abiotic variables measured and in the (a) class (b) order, (c) family, and (d) ESV CO1 community composition across the three different habitat types in the MNWR, using stepwise sequential tests following AICc selection criterion (Prop. var. = proportion of variation out of total variation).

(a) Class				
Marginal test	SS (trace)	Pseudo-F	*p* value	Prop. var
C	311.8	0.435	0.860	0.0264
N	305.44	0.42589	0.866	0.0259
C:N ratio	315.92	0.4409	0.861	0.0268
NH_4_^+^	673.36	0.97001	0.441	0.0571
NO_3_^−^	546.66	0.7786	0.567	0.0464
C_mic_	311.03	0.4339	0.870	0.0264
pH	368.69	0.51693	0.773	0.0312
Moisture	2602	4.5358	0.002	0.2209

Sequential test	AICc	Pseudo-F	*p* value	Prop. var
Moisture	117.02	4.5358	0.002	0.2209
pH	116.89	2.7586	0.012	0.1210

(b) Order				
Marginal test	SS (trace)	Pseudo-F	*p* value	Prop. var
C	709.72	0.51222	0.959	0.0310
N	849.77	0.61719	0.89	0.0371
C:N ratio	819.8	0.59462	0.900	0.0358
NH_4_^+^	1594.7	1.1988	0.245	0.0697
NO_3_^−^	1887.7	1.4388	0.125	0.0825
C_mic_	2065.6	1.5879	0.084	0.0902
pH	1000	0.73129	0.769	0.0437
Moisture	3261.6	2.6602	**0.003**	0.1425

Sequential test	AICc	Pseudo-F	*p* value	Prop. var
Moisture	130.69	2.660	0.006	0.1426

(c) Family				
Marginal test	SS (trace)	Pseudo-F	*p* value	Prop. var
C	1299.6	0.92057	0.526	0.0544
N	1494.2	1.0676	0.39	0.0625
C:N ratio	372.87	0.25371	0.995	0.0156
NH_4_^+^	1000.7	0.69955	0.789	0.0418
NO_3_^−^	1363.3	0.96838	0.481	0.0570
C_mic_	1464.3	1.0448	0.379	0.0612
pH	1549.9	1.1101	0.339	0.0648
Moisture	3359.4	2.6183	**0.004**	0.1406

Sequential test	AICc	Pseudo-F	*p* value	Prop. var
Moisture	131.51	2.618	0.004	0.1406

(d) ESV				
Marginal test	SS (trace)	Pseudo-F	*p* value	Prop. var
C	2938.1	0.77698	0.986	0.0463
N	3151.1	0.83628	0.948	0.0496
C:N ratio	2556.4	0.67183	0.998	0.0402
NH_4_^+^	4449.6	1.2069	0.076	0.0701
NO_3_^−^	3912.5	1.0516	0.291	0.0616
Cmic	4535.4	1.2319	0.068	0.0714
pH	4292.3	1.1611	0.111	0.0676
Moisture	5121.7	1.4052	**0.008**	0.0807

Sequential test	AICc	Pseudo-F	*p* value	Prop. var
Including moisture	150.3	1.4052	0.016	0.0807
Excluding moisture	149.26	1.4052	0.011	0.0807

**Table 6:** A distance-based linear model (DistLM) of the marginal and sequential tests of the relationship between the soil abiotic variables measured and in the (a) class (b) order, (c) family, and (d) ESV CO1 Richness across the three different habitat types in the MNWR, using stepwise sequential tests following AICc selection criterion. Significant results are indicated by *p* < 0.05 in bold (Prop. var. = proportion of variation out of total variation).

(a) Class				
Marginal test	SS (trace)	Pseudo-F	*p* value	Prop. var
C	0.07456	0.33998	0.590	0.02080
N	0.00414	0.018	0.902	0.00115
C:N ratio	0.27484	1.329	0.262	0.07669
NH_4_^+^	0.02535	0.11403	0.746	0.00707
NO_3_^−^	0.42989	2.181	0.166	0.11996
Cmic	1.0772	6.8766	0.019	0.30060
pH	0.35968	1.7851	0.224	0.10037
Moisture	0.18601	0.87596	0.384	0.05190

Sequential test	AICc	Pseudo-F	*p* value	Prop. var
C_mic_	− 30.69	6.8766	0.019	0.3006

(b) Order				
Marginal test	SS (trace)	Pseudo-F	*p* value	Prop. var
C	0.32754	0.082982	0.76	0.00515
N	0.12232	0.03089	0.868	0.00192
C:N ratio	0.14107	0.035635	0.869	0.00222
NH_4_^+^	0.49489	0.12571	0.72	0.00779
NO_3_^−^	6.8064	1.9215	0.197	0.10722
C_mic_	32.837	17.145	0.002	0.51727
pH	9.3556	2.7656	0.119	0.14738
Moisture	0.48127	0.12223	0.738	0.00758

Sequential test	AICc	Pseudo-F	*p* value	Prop. var
C_mic_	14.38	17.15	0.004	0.5173

(c) Family				
Marginal test	SS (trace)	Pseudo-F	*p* value	Prop. var
C	0.00810	0.018743	0.896	0.00117
N	0.03812	0.088524	0.777	0.00550
C:N ratio	0.01803	0.041762	0.812	0.00260
NH_4_^+^	0.09211	0.21558	0.652	0.01329
NO_3_^−^	1.3458	3.8568	0.074	0.19423
C_mic_	1.6218	4.8896	0.039	0.23407
pH	0.30376	0.73359	0.411	0.04383
Moisture	0.14431	0.34033	0.546	0.02082

Sequential test	AICc	Pseudo-F	*p* value	Prop. var
C_mic_	− 17.18	4.89	0.048	0.2341

(d) ESV				
Marginal test	SS (trace)	Pseudo-F	*p* value	Prop. var
C	5.0305	0.035258	0.858	0.00219
N	6.7294	0.04720	0.837	0.00294
C:N ratio	0.068556	0.000479	0.986	0.00002
NH_4_^+^	0.011711	0.000081	0.995	0.00001
NO_3_^−^	32.606	0.23132	0.629	0.01425
C_mic_	831.63	9.1371	0.006	0.36349
pH	457.56	3.9999	0.062	0.19999
Moisture	3.7867	0.026526	0.867	0.00165

Sequential test	AICc	Pseudo-F	*p* value	Prop. var
C_mic_	83.88	9.14	0.01	0.3635

## Discussion

Many soil invertebrate studies examining community structure typically rely on traditional morphological-based tools to assign identity to soil invertebrates captured using Berlese funnel extraction or pitfall traps. However, recent DNA metabarcoding advances have allowed us to use marker gene surveys as a tool to assess community ecology-based questions and is becoming increasingly popular for soil-based invertebrate studies^42^. In this study, we amplified 3 fragments of the standard 5′ end of the 658 bp mitochondrial CO1 region and DNA metabarcoding to assess soil invertebrate community composition and richness at four different taxonomic resolutions across a primary forest and two different ages of secondary forest, and the associated abiotic drivers with community composition and richness. Even though there are current reference database limitations in assigning invertebrate taxonomy, it is clear from this study that community-level differences in soil invertebrate composition and the associated soil abiotic drivers are detectable via DNA metabarcoding from bulk-soil across a land management gradient in the MWNR of Costa Rica.

### Soil invertebrate community composition

Overall, the results showed that soil invertebrate community composition is different, and even 33 years of natural regrowth can have consequences on the soil invertebrate community that develops under these conditions and can result in community-level differences across primary and secondary forests. This is not surprising as previous evidence has shown that land use history or subsequent secondary growth can create changes and differences in the soil decomposer communities that develop under these conditions due to changes in resource input^42^. Moreover, a study using DNA metabarcoding of arthropod orders showed that certain groups of invertebrates were significantly different across land use types in southwest China^43^. In terms of taxonomic resolution, the soil invertebrate community composition at the ESV level was distinctly different across the primary and secondary forests, compared to differences at class, order, and family resolutions. This finding is most likely due to finer differences that can be detected at a greater taxonomic resolution in the data-set at the ESV level, and has been described elsewhere^44,45^.

Past land use history is known to influence the vegetation type observed in secondary forests. Ultimately, the changes in vegetation type between pre- and post-disturbance and likely soil disturbance can affect the types of substrates entering the soil through decomposition of leaf litter, as well as changes in the soil chemistry and soil nutrient and organic matter content. Indeed, fluctuations in soil characteristics can affect the abundance and distribution of soil biotic communities and often depends on the individual tolerance limits of different soil invertebrates at the local and regional scale^46–49^. We found that changes in soil invertebrate community composition were best explained by soil moisture at the class, order, and family level. However, the percent soil moisture accounted for relatively little of the total variation (up to 22%) in the soil invertebrate community structure, indicating that there could be an additional unknown soil environmental variable driving the variation observed. Previous studies have shown certain groups of soft-bodied invertebrates, such as collembola and some groups of mites to be effected by varying levels of moisture or water content^47,50–53^. Even though percent soil moisture was not significantly different between the three habitats, it could be that subtle fluctuations in soil moisture not detected here can influence particular soil invertebrate groups. The most prevalent invertebrate families detected in the soils across sites were Enchytraeidae, Naididae, Megascolecidae, Isotomidae, Entomobryidae, Onychiuridae and are invertebrate families that prefer moist conditions^48^. Thus, these families with the greatest representation across the primary and secondary forest soils are likely to be highly sensitive to changes in soil moisture, as demonstrated by differences in relative abundance and distribution of these groups across land-use.

A general model of soil invertebrate responses to past and/or current land use history is difficult to define. However, by examining and detecting differences in the soil invertebrate community in conjunction with the associated soil abiotic drivers, we can begin to assess the influence of past and present land use on soil-dwelling invertebrates. We have demonstrated in this study that soil invertebrate community composition changes can be detected, even over the relatively short time frame of 33 years natural forest regrowth. The outcomes and trajectories of these altered invertebrate communities are still largely unknown and warrants further investigation. It is likely that similar transitions of soil invertebrate communities, depending on land use and stage of reforestation, are currently occurring across the globe, and we have demonstrated that DNA metabarcoding can be applied to determine both the extent of community shifts and the associated abiotic drivers of the soil invertebrate structure.

### Soil invertebrate richness and diversity

While we did not find differences in soil invertebrate Shannon diversity at all taxonomic levels across the primary and two secondary forests, we did find that soil invertebrate richness was greatest in the primary forest soils with lower richness in both of the secondary forests. This pattern may indicate that secondary forest developments may not be able to sustain the soil invertebrate richness to the same extent as the primary forest, even after 33 years of regrowth. This is most likely due to a primary forest presumably having higher diversity, complexity and richness of vegetation than resulting secondary forest growth, which has been described previously at this site. A greater multiplicity of resources and spatial heterogeneity (such as greater quantity, quality, and complexity of plant litter) on the forest floor in primary forests would presumably create additional niches or a partitioning of resources, resulting in a greater richness of soil invertebrate communities present to consume these resources^55,69^.

Previous evidence suggests there is a strong link between soil arthropod biomass and soil microbial biomass, and that soil arthropod biomass is typically regulated by bottom-up forces^28^. To corroborate this, we found that as soil microbial biomass C increases, soil invertebrate richness also increases, and that soil C_mic_ explained up to 52% of the total variation observed in the soil invertebrate richness. Soil C_mic_ is a measure of the C contained within the living component of soil organic matter and as microbial organisms decompose soil organic matter, this releases carbon dioxide and plant nutrients that become available to the surrounding biota. Thus, as soil organic matter increases, so should soil C_mic_, and thus, more soil organic C available to biotic communities. However, soil C_mic_ is strongly correlated with the amount and availability of soil organic matter, and is also influenced by soil properties such as pH, soil type and texture (i.e. percent sand, silt, and clay)^48,54^. Therefore, land use activities that cause reductions of essential soil C and N through the loss in the amount and complexity of vegetation and leaf litter, thereby decreasing the availability of soil organic matter, will also decrease the amount of soil C_mic_, and consequently, will also cause a decrease in the soil invertebrate richness. As a previous study determined that at our study site, the soil texture was uniform across the three forest types^55^, we can therefore assume that shifts in microbial biomass, as a consequence of land use and afforestation status, are likely to affect soil fauna assemblages observed. We have demonstrated that previous land use histories and current practices that remove or reduce soil microbial biomass C could have major implications for the composition of soil invertebrate communities, which directly influences the extent of ecological functions being performed across different land management gradients.

### Taxonomic contributions

The SIMPER analysis revealed that the taxonomic groups contributing the most to the dissimilarity between habitats were those invertebrates involved with processing plant and soil material. These invertebrates are microbivores that feed on bacteria and fungi, either directly or indirectly, and contribute to the available soil organic matter content. The most prevalent microbivore invertebrate orders were Haplotaxida and Entomobryomorpha, or more specifically, the families Enchytraeidae, Naididae, Onychiuridae, Elateridae, and Isotomidae. When interpreting SIMPER results, it is important to take into account the proportion data; for example, despite Enchytraeidae being one of the top taxonomic groups contributing to the percent dissimilarity across habitats, this invertebrate family also had the greatest representation in the 33-year-old secondary forest. This could reveal a more hump-backed or bell-shaped response in relation to succession. Ideally, a good soil invertebrate indicator should have an increasing trend in the representation of the invertebrate group across succession, to provide an indication if secondary forest soils are on a trajectory to that of a primary forest.

In accordance with the proportion data, Naididae, Entomobyridae, and Elateridae, show an increasing trend of representation from young and old secondary forest to primary forest. The observation that the primary forest soils had a greater representation of these three invertebrate families suggests the importance of these groups as potential terrestrial indicators in this area for understanding the future trajectory and recuperation of soil in these secondary forests. The larvae of the Elateridae are (known as ‘wireworms’) live in soil and/or under bark and feed and process decaying soil organic matter^56^. The wireworm larval stage can span from 2 to 6 years, and in some cases even 10 years^56–58^. During this larval period, wireworms specifically feed underground making them persistent residents of soil for some time. Therefore, the Elateridae detected in this study could most likely be in the larval stage and may be an over-looked group in tropical soils of this area. Although wireworms are often agricultural and garden pests by feeding on plant tissue and roots, in this study the wireworms may represent an important fraction of the soil invertebrate community involved in the recycling of decaying organic matter^57^. These decomposition-based activities can increase the amount and availability of resources to the surrounding soil biotic communities. Wireworms are known to be influenced by soil characteristics such as soil texture^57^. However, given that these forests are of the same soil texture, the greater representation of Elateridae in the primary forest than both of the secondary forests may be an indicator of more soil organic matter and decaying materials for substrates for the wireworms.

The greater representation of Naididae in primary forest soils could indicate a higher substrate availability to enable the build-up of soil organic matter for plant nutrient acquisition and growth of soil organisms. Naididae are important oligochaetes capable of increasing the availability of soil N through bioturbation and feeding activities^59–61^. For example, Naididae excrete NH_4_^+^ by ingesting silt and clay particles saturated with microbial groups attached to the soil particles^60^. These feeding activities could be an important avenue for increasing soil N, particularly where soil N has been depleted following agricultural abandonment or the early stages of tropical lowland secondary forests^62–65^.

Most of the groups present in the SIMPER results consist of groups from the soil mesofauna. Entomobyridae are a group of collembola and make-up an important fraction of the mesofauna. Indeed, collembolan groups have been used as indicators of soil condition in other landscapes, but less is known about this group in secondary forests of the tropics. Different groups of collembolans have different feeding preferences, and can suggest different scenarios regarding their presence or absence from an ecosystem^48^. For example, collembolan groups Isotomidae and Onychiuridae were contributing taxonomic groups in the SIMPER results; however, Isotomidae and Onychiuridae had greater representation in the secondary forest soils than the primary forest soils. This suggests that different groups of collembolans might show greater representation during different periods of succession, but that collembolan groups such as Entomobryidae may be an indication of late succession or even a non-disturbed habitat such as primary forest soils. Entomobryidae collembolans could be a potential indication as to whether or not secondary forests and soils are approaching a state close to the primary forest soils.

Taken together, these findings indicate that previous land-use history and subsequent regrowth can influence particular groups of soil invertebrates that can be used as potential terrestrial indicators of succession in the tropics.

## Conclusions

In the present study, we determined if soil invertebrate community composition at class, order, family, and ESV level was different between a primary forest, 33-year-old secondary forest, and a 23-year-old secondary forest with the same soil type and texture but with differing past land-use histories and which soil environmental variables were structing the community composition and richness. Our work suggests that past land-use history and associated forest regrowth can lead to shifts in the structure of soil invertebrate community, but differences are more prominent or detectable at finer resolutions such as the ESV level. Moreover, these past land-use histories and subsequent secondary forest developments can alter soil invertebrate richness but not diversity, such that the soil invertebrate richness has not reached or returned to pre-disturbed state. These findings were all detectable with DNA metabarcoding of the CO1 region, providing a useful and rapid approach in understanding soil invertebrate biodiversity across succession in the tropics. Nonetheless, using DNA metabarcoding to analyze soil invertebrates at the community-based scale, as opposed to specific lineages, it is possible to detect changes in the structure of the soil invertebrate community even across landscapes of the same soil texture but managed differently.

As global, and in particular tropical deforestation continues today, and as the recuperation of degraded soils is becoming increasingly important, it is crucial to understand which soil abiotic factors most strongly drive soil invertebrate community dynamics, with implications for how to best improve soil conditions. This study provides valuable knowledge and applicability in using DNA metabarcoding to detect community-level differences in soil invertebrate structure from bulk-soil across a land management gradient and subsequent forest development. Using rapid techniques such as DNA metabarcoding will become increasingly invaluable and a potentially more powerful technique to capture changes in soil invertebrate communities under land use changes.

## Methods

Due to legal and illegal extraction-based land management practices since the 1970’s, the San Juan-La Selva Biological Corridor (SJLBC) (Fig. S1) was created in 2001 by the Costa Rican Ministry of Environment and Energy to protect 1,204,812 hectares of ecosystems for habitat connectivity and biodiversity in the Northern Zone of Costa Rica^66^. Within the SJLBC, the Maquenque National Wildlife Refuge (MNWR) (10° 27′ 05.7″ N, 84° 16′ 24.32″ W) was established in 2005 by executive decree of the Costa Rican government to protect over 50,000 hectares of various ecosystems (Fig. S1). The MNWR is the core nucleus of the SJLBC for biodiversity as it contains the highest percentage of forest cover and most valuable habitats for biodiversity in the region^66^. Mean annual temperature is 27 °C, mean annual rainfall is 4300 mm, and the dominant soil type are oxisols^67^.

This study was conducted in the humid Atlantic lowland rainforests of the MNWR in three upland habitat types that were at one point in time all a part of a single tract of primary forest, with the same soil type (oxisol), texture^55^, and topography, but have been managed differently in the past ~ 40 years (total area 500 hectares; personal communication). The resulting habitats consist of a primary forest that has not been harvested in the known history of the land; a 33-year-old secondary forest that was allowed to regenerate immediately following deforestation (harvested 33 years ago); and a 23-year-old secondary forest that was cut 33 years ago, used for cattle pasture for ~ 10 years, and then allowed to regenerate into secondary-growth. In July 2014, 6 independent replicate plots (40 m × 25 m) were established in each of the three habitats. Distance between forest sites are as followed: primary forest (10° 40′ 46.21″ N, 84° 10′ 42.10″ W) and young secondary forest (10° 41′ 7.92″ N, 84° 9′ 57.30″ W) is ~ 1.5 km, primary forest and old secondary forest (10° 41′ 12.56″ N, 84° 10′ 15.65″ W) is ~ 1.1 km, old and young secondary forest is ~ 0.55 km. In each 1000 m^2^ plot, nine soil subsamples were collected using a soil core (7.5 cm × 15 cm ×   1.25 cm) in a grid fashion (Fig. S2) from each plot and bulked to provide one composite soil sample for each replicate plot (6 plots × 3 habitats = 18 bulked independent samples). The soil core and soil collection gloves were sterilized with 70% ethanol between each 1000 m^2^ replicate plot to minimize cross-contamination and ensure independence of soil samples. This approach to soil sampling was to reduce microsite variability given the heterogeneous properties in tropical soils^68^. The soil pH and percent moisture were recorded during the same time of soil sample collection at each soil profiler core sample location in each of the plots in triplicate readings (3 readings × 9 soil core locations = 27 readings per plot) (Fig. S1) with a Kelway Soil pH and Moisture meter (Kel Instruments Co., Inc., Wyckoff, NJ, USA). Elevation of these plots were measured with a Garmin Rino 650 GPS (Garmin International, Olathe, KS, USA). For homogenization, all soil samples were mixed and passed through a 4-mm sieve at field moist conditions while sterilizing the sieve and laboratory gloves with 70% ethanol between each of the 18 composite soil samples, prior to all downstream analyses.

Soil abiotic factors have been measured previously and described elsewhere^55^. The soil abiotic properties examined in this study for each of the three habitats included soil pH, % moisture, C, N, C:N ratio, NH_4_^+^, NO_3_^−^, C_mic_, and % sand, silt, and clay. As aforementioned, soil pH and % moisture were measured from each of the 18 plots during the period of soil subsample collection To estimate (ToC), (TN), NH_4_^+^, and NO_3_^−^, 200 g of soil from each of the 18 sieved composite soil samples were delivered to the Center for Tropical Agriculture Research and Education (CATIÉ) Laboratories in Turrialba, Costa Rica. To estimate levels of microbial biomass C (C_mic_), substrate-induced respiration (SIR) was used following the methods of Höper (2006). Substrate-induced respiration for measuring C_mic_ was preferred as it measures the amount of living microbial biomass C. All nutrient and microbial activity data presented were adjusted for dry weight of the soil.

Environmental DNA was extracted from each of the 18 composite soil samples using three 0.33 g replicate sub-samples for a total of 1 g for each composite soil sample using the MoBio PowerSoil DNA Isolation Kit (MO BIO Laboratories Inc., Carlsbad, CA, USA) and according to the manufacturer’s protocol. The concentration and purity (A_260_/A_280_ ratio) of extracted soil DNA were determined prior to downstream analyses using a NanoDrop 1000 spectrophotometer (ThermoFisher Scientific, Waltham, MA) and all eDNA was stored at − 80 °C, as has been done in previous studies^55,69,70^.

Three fragments of the standard 5' end of the 658 bp mitochondrial cytochrome c oxidase I gene (CO1) were amplified targeting three fragments: F230 (~ 230 bp), BR (~450 bp), and BR5 (~ 330 bp) using three primer sets (LCO1490: 5′-GGTCAACAAATCATAAAGATATTGG-3′^71^ and 230_R: 5′-CTTATRTTRTTTATICGIGGRAAIGC-3′^44^, B: 5′-CCIGAYATRGCITTYCCICG-3′^72^ and R: 5’-TAAACTTCAGGGTGACCAAAAAATCA-3’^71^; and ArR5, 5′-GTRATIGCICCIGCIARIACIGG-3′^73^. Amplicons were prepared with two-steps PCR regime. The first PCR used the CO1 specified primers and the second PCR involved Illumina-tailed primers. Each PCR amplification contained of 2 µL DNA template, 17.5 µL molecular biology grade water, 2.5 µL 10 × reaction buffer (200 mM Tris-HCl, 500 mM KCl, pH 8.4), 1 µL 50 × MgCl_2_ (50 mM), 0.5 µL of dNTPs mix (10 mM), 0.5 µL of forward primer (10 mM), 0.5 µL of reverse primer (10 mM), and 0.5 µL of Invitrogen’s Platinum Taq polymerase (5 U/µL) in a total volume of 25 µL. The PCR conditions were initiated with heated lid at 95 °C for 5 min, followed by a total of 35 cycles of 94 °C for 40 s, 46 °C (for all primer sets) for 1 min, and 72 °C for 30 s, and a final extension at 72 °C for 5 min, and hold at 4 °C. PCR products were then purified using a Qiagen MinElute PCR purification kit (Qiagen, Valencia, CA, USA) and eluted in 30 µL of molecular biology grade water. A second PCR step was implemented using the purified 1st PCR product as a template and with Illumina adaptor tailed target specific primers. The 2nd PCR was made following the same protocol as aforementioned except for 30 cycles were used for PCR. All PCR products were visualized on 1.5% agarose gels to confirm successful amplification by the presence of fluorescent bands, as has been done previously^55,69,70^. All PCRs were done using Eppendorf Mastercycler ep gradient S thermalcyclers and negative control reactions (no DNA template) were included in all experiments, and all generated soil amplicons plates were dual indexed and pooled into a single tube and sequenced in several Illumina MiSeq runs using a V2 MiSeq sequencing kit (500 cycles—250 × 2) (FC-131-1002 and MS-102-2003).

The raw Illumina paired-end reads were processed using the SCVUC v2.3 pipeline available from https://github.com/EcoBiomics-Zoobiome/SCVUC_CO1_metabarcode_pipeline. The subsequent CO1 Illumina generated sequences were processed using semi-automated pipelines. The CO1 Illumina paired-end libraries generated forward (R1) and reverse (R2) reads. Raw sequence reads were paired with SeqPrep ensuring a minimum Phred score of 20 and minimum overlap of at least 25 bp^74^. Primer sequences were trimmed using CutAdapt v1.10 specifying a minimum Phred score of 20, a minimum fragment length of 150 bp after trimming, and no more than 3 N’s allowed. Reads were dereplicated with VSEARCH v2.11.0 using the ‘derep_fulllength’ command and the ‘sizein’ and ‘sizeout’ options. Denoising was performed using the UNOISE3 algorithm in USEARCH v10.0.240^75^. This method corrects sequences with potential errors, removes putative chimeric sequences, as well as removes rare exact sequence variants (ESVs)^76^ with only one or two reads (singletons and doubletons). Previous work has shown that rare sequence clusters may be particularly prone to sequence errors and add ‘noise’ to the dataset^77,78^. Here we defined rare reads to be ESVs containing only 1 or 2 reads (singletons and doubletons). An ESV x sample table was created with VSEARCH using the ‘usearch_global’ command, mapping reads to ESVs with 100% identity. The remaining ESVs were taxonomically assigned using the CO1 Classifier v3.2^41^.

All generated sequencing data have been deposited in the National Center for Biotechnology Information (NCBI) Sequence Read Archive (SRA) database under the BioProject: PRJNA542652.

A total of 1,117,015 ESV reads were taxonomically assigned by the CO1 classifier^41^. After filtering out the reads identified as bacteria, fungi, plants, and non-metazoan, 534,185 reads were retained. Out of the 534,185 reads, 117,162 reads were correctly identified to class and order at the 0.0 bp cutoff for 100% accuracy as is recommended for 200 bp fragments^41^. At the family level, 38,690 reads were correctly identified at the 0.20 bp cutoff, and at genus level, only 4,753 reads were correctly assigned at the 0.30 bp cutoff for 99% accuracy^41^. Thus, the soil invertebrate reads used in further analyses were class, order, family, and ESV levels. All CO1 ESVs were organized taxonomically at the class (0.0 bp cutoff), order (0.0 bp cutoff), family (0.2 bp cutoff), and ESV rank, and converted to mean proportion of sequences per sample^79^. The relative proportion of CO1 sequences for class, order, family, and ESVs were calculated by summing the number of reads for each taxon in a sample, then dividing by the total number of reads from the sample (i.e. sample replicate). To examine the soil CO1 class, order, family, and ESV richness and diversity for each sample, Margalef’s richness (d = (S − 1)/Log(N)) and Shannon Index (*H′*) (*H′* = −SUM(P_i_*ln(P_i_))) (P_i_ is the proportion of the total count (abundance) arising from the *i*th class; order; family; ESV) were calculated in PRIMER-E v6^80^. Soil CO1 class, order, family, and ESV community compositions were transformed using a square-root transformation to account for dominant taxa as well as rare taxa^80^. For a weighted approach, the square-root transformed CO1 data were then calculated into a Bray–Curtis dissimilarity matrix in PRIMER-E v6^80^.

### Statistical and multivariate analyses

To address question one, significant differences in the means of the alpha diversity indices for soil CO1 class, order, family, and ESVs across the primary forest and secondary forests were determined using a one-way ANOVA followed by Tukey’s HSD post-hoc analyses in SPSS (v.25, Armonk, NY, USA). Prior to analyses, a Shapiro–Wilk test was performed to determine normality of all the data in SPSS (v.25, Armonk, NY, USA) and all data were *p* > 0.05 suggesting normality. To determine if there were differences in the Bray–Curtis dissimilarity soil CO1 class, order, family, and ESV community composition matrices between the three forests, a one-way permutational multivariate analysis of variance (PERMANOVA) with main and pair-wise tests based on unrestricted permutations (999 permutations)^81^. The PERMANOVA results of the soil CO1 community compositions across the primary and secondary forests were considered significant if *p* ≤ 0.05.

In addition, a Canonical Analysis of Principal Coordinates (CAP) was used to visualize the distinctness of the soil CO1 class, order, family, and ESV community compositions across the primary forest and secondary forest soils based on an a priori allocation success using the PERMANOVA + guidelines^80,82^. Strong differences between primary forest and secondary forest soils are represented by CAP axis 1 and CAP axis 2 squared canonical correlations greater than or equal to 0.7, and moderate differences are represented by squared canonical correlations greater than or equal to 0.5–0.69^80,82^. Furthermore, Cohen’s *d* effect sizes were calculated for the PERMANOVA pairwise comparisons to assess if the differences were trivial or not, and used as indicators of biologically meaningful differences between mean values of the parameters measured, as recommended for analysis of small sample sizes^83^. Cohen’s *d* effect size statistics are considered small if *d* = 0.2, medium if *d* = 0.5–0.7, and large if *d* > 0.8.

To address question two, a Similarity of Percentage analysis (SIMPER) was used to determine which CO1 class, order, and family taxonomic groups primarily contribute to the Bray–Curtis dissimilarity between the habitat pairwise groups using a one-way design with a 70% cutoff percentage to list only higher-contributing taxonomic groups^84^. The SIMPER analysis was performed to potentially indicate or elude to the higher contributing taxonomic groups whose changes in proportion of sequences may indicate change; not that these groups are responsible for the biological dissimilarity in community composition across these forests.

To address question three, a Distance-Based Linear Model approach (a multivariate multiple regression) was implemented to determine which soil environmental variable is contributing the most to the dissimilarities between habitats. The DistLM was performed using a ‘step-wise’ selection procedure and an AICc (Akaike’s Information Criterion Corrected) selection criterion with 999 permutations^80,85^. The Distance-Based Linear Modeling sequential tests were considered significant if *p* ≤ 0.05. These DistLM results were then visualized using a Distance-Based Redundancy Analysis (dbRDA) in which the ordination is plotted from the values of the given model that explains the greatest variation in the data cloud^86^.

All multivariate analyses were performed in PRIMER-E v6^84^ and its add-on PERMANOVA+^80,82^. Prior to multivariate analyses, draftsman plots (variable pair-wise scatter plots) were used to determine the homogeneity and multicollinearity of each soil abiotic variable (predictor variables). All soil abiotic variables were transformed using the log (x + 1) transformation to correct for skewness and the data standardized using the ‘normalize’ parameter in PRIMER-E v6^80,82,84^.

## Supplementary information

Supplementary Information.
