# Supplementary material for: Drivers of tropical soil invertebrate community composition and richness across tropical secondary forests using DNA metasystematics
Source: Sci Rep. 2020 Oct 28;10:18429. doi: 10.1038/s41598-020-75452-4 (PMC7595130; doi:10.1038/s41598-020-75452-4)
Supplement: Supplementary file 1 — Supplementary Information. [file 41598_2020_75452_MOESM1_ESM.docx]

Drivers of tropical soil invertebrate community composition and richness across tropical secondary forests using DNA metasystematics

Katie M. McGee^1*^, Teresita M. Porter^1^, Michael Wright^1^, M­ehrdad Hajibabaei^1^

^1^Centre for Biodiversity Genomics at Biodiversity Institute of Ontario and Department of Integrative Biology, University of Guelph, 50 Stone Road East, Guelph, ON, N1G 2W1, Canada

*Corresponding author: Katie M. McGee, Fax: +1 519 824 5703

Email: kmcgee@uogueph.ca

**Supplementary material**

**
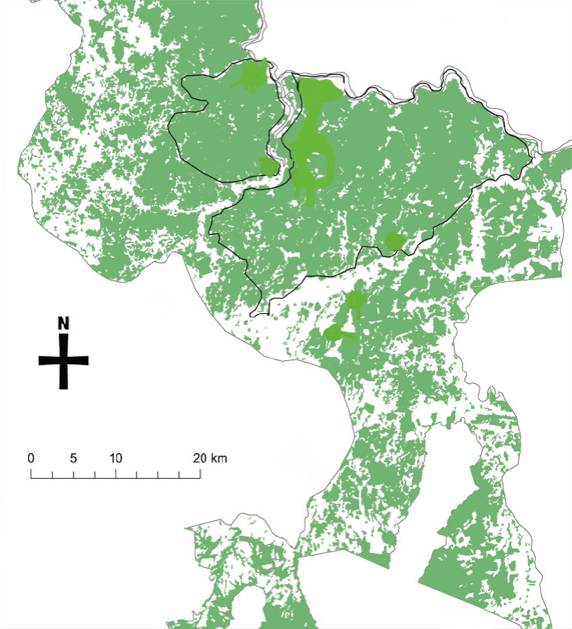

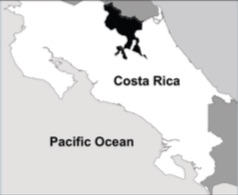
**

**Fig. S1** Map of the (left) San Juan La-Selva Biological Corridor (shaded in black) and the (right) Maquenque National Wildlife Refuge (MNWR in green outlined in black).

**Fig. S2** Soil core sampling design schematic for the 1,000 m^2^ replicate plots in each habitat type within the MNWLR. Nine soil cores (green rectangles) were collected and bulked in one bag for one composite soil sample for each plot. In each soil core location triplicate soil *p*H and percent moisture readings were taken.

**Table S1.** Mean values and standard errors of the soil abiotic properties across a primary forest, an old (33-years-old) secondary forest, and a young (23-years-old) secondary forest, and the one-way ANOVA results evaluating the different soil abiotic properties across these three habitats in the Northern Zone of Costa Rica. Different letters denote significant pairwise comparisons (*p* < 0.05) based on post-hoc analyses (McGee et al., 2018).

| Soil Abiotic Property | Primary | Old Secondary | Young Secondary | ANOVA | | |
| --- | --- | --- | --- | --- | --- | --- |
|  |  |  |  | d.f. | F stat | *p*-value |
| C (%) | 5.83 ± 0.21 | 6.16 ± 0.35 | 5.69 ± 0.18 | 2,15 | 0.867 | 0.440 |
| N (%) | 0.47 ± 0.02 | 0.48 ± 0.02 | 0.45 ± 0.01 | 2,15 | 1.893 | 0.185 |
| C:N | 12.45 ± 0.16 | 12.44 ± 0.55 | 12.60 ± 0.11 | 2,15 | 0.066 | 0.936 |
| NO_3_^-^ (µgN/g) | 31.55 ± 8.07 | 34.51 ± 2.87 | 23.20 ± 4.88 | 2,15 | 1.061 | 0.371 |
| NH_4_^+^ (µgN/g) | 11.86 ± 0.69^a^ | 9.10 ± 0.47^b^ | 14.74 ± 1.16^ac^ | 2,15 | 11.751 | 0.001 |
| C_mic_ (µgC/g) | 851.92 ± 94.05^a^ | 614.09 ± 19.22^ab^ | 532.24 ± 36.84^bc^ | 2,15 | 7.825 | 0.005 |
| pH | 5.70 ± 0.03 | 5.89 ± 0.05 | 5.82 ± 0.08 | 2,15 | 3.135 | 0.073 |
| Moisture (%) | 56.72 ± 1.16 | 59.72 ± 1.95 | 53.97 ± 3.37 | 2,15 | 1.451 | 0.265 |
| Elevation (m) | 58.75 ± 0.59 | 58.93 ± 0.41 | 59.92 ± 1.72 | 2,15 | 0.343 | 0.715 |
| Sand (%) | 55.56 ± 0.62 | 54.92 ± 1.41 | 57.03 ± 1.03 | 2,12 | 1.023 | 0.389 |
| Silt (%) | 5.91 ± 0.88 | 6.10 ± 0.69 | 6.89 ± 0.69 | 2,12 | 0.469 | 0.636 |
| Clay (%) | 41.50 ± 1.27 | 40.0 ± 0.67 | 39.56 ± 2.37 | 2,12 | 0.404 | 0.676 |

**Table S2.** COI class proportion of sequences greater than 1%

| **Class** | **Primary** | **Old Secondary** | **Young Secondary** |
| --- | --- | --- | --- |
| Clitellata | 22.4988 | 48.9432 | 14.5616 |
| Insecta | 30.6732 | 24.9377 | 21.0466 |
| Collembola | 19.1567 | 3.2123 | 28.3412 |
| Arachnida | 10.8713 | 5.5153 | 17.8634 |
| Chromadorea | 9.8300 | 4.1655 | 5.3960 |
| Polychaeta | 0.6159 | 5.0755 | 5.9399 |
| Chilopoda | 0.8365 | 4.6633 | 0.5128 |
| Malacostraca | 1.7944 | 1.2544 | 1.8981 |
| Gastropoda | 1.6638 | 0.7189 | 2.2374 |
| Cestoda | 1.4566 | 0.2197 | 1.2846 |
| Trematoda | 0.2260 | 0.1873 | 0.2285 |
| Maxillopoda | 0.0336 | 0.3456 | 0.0771 |
| Diplopoda | 0.1318 | 0.1747 | 0.1263 |
| Pycnogonida | 0.0022 | 0.1590 | 0.2306 |
| Heterotardigrada | 0.0507 | 0.0000 | 0.2560 |
| Udeonychophora | 0.0000 | 0.2145 | 0.0000 |
| undef_Platyhelminthes | 0.0873 | 0.0889 | 0.0000 |
| undef_Gastrotricha | 0.0448 | 0.0714 | 0.0000 |
| Eutardigrada | 0.0000 | 0.0527 | 0.0000 |
| Palaeonemertea | 0.0116 | 0.0000 | 0.0000 |
| Cephalocarida | 0.0057 | 0.0000 | 0.0000 |
| undef_Annelida | 0.0036 | 0.0000 | 0.0000 |
| Anopla | 0.0033 | 0.0000 | 0.0000 |
| Monogenea | 0.0022 | 0.0000 | 0.0000 |

**Table S3.** COI order proportion of sequences greater than 1%

| **Order** | **Primary** | **Old Secondary** | **Young Secondary** |
| --- | --- | --- | --- |
| Haplotaxida | 22.4470 | 48.8905 | 14.2038 |
| Entomobryomorpha | 17.2021 | 3.0921 | 26.4398 |
| Coleoptera | 11.3763 | 2.8190 | 1.7546 |
| Ascaridida | 7.8369 | 3.1835 | 4.5340 |
| Sarcoptiformes | 0.6236 | 1.0886 | 11.3084 |
| Hemiptera | 1.5697 | 3.4324 | 6.2703 |
| Lepidoptera | 0.8123 | 6.7572 | 3.3075 |
| Araneae | 3.2667 | 2.9601 | 4.5972 |
| Blattodea | 7.7094 | 0.3793 | 2.2808 |
| Odonata | 3.2403 | 2.7499 | 4.3690 |
| Phyllodocida | 0.4716 | 2.9642 | 4.2590 |
| Diptera | 2.4211 | 3.1152 | 0.8018 |
| Ixodida | 4.3879 | 0.1475 | 0.0782 |
| Hymenoptera | 0.6250 | 2.2938 | 1.4912 |
| Decapoda | 1.4229 | 1.0588 | 1.7807 |
| Scorpiones | 2.2992 | 0.7171 | 1.1025 |
| Poduromorpha | 1.9529 | 0.1202 | 1.9014 |
| Scolopendromorpha | 0.0764 | 3.2119 | 0.5128 |
| Stylommatophora | 1.5036 | 0.4918 | 1.6980 |
| undef_Polychaeta | 0.0000 | 1.3571 | 1.4576 |
| Ephemeroptera | 1.8422 | 0.3384 | 0.0000 |
| Phasmatodea | 0.5487 | 0.8312 | 0.4099 |
| Rhabditida | 1.0629 | 0.1936 | 0.3317 |
| Orthoptera | 0.0674 | 1.4402 | 0.0000 |
| Geophilomorpha | 0.0000 | 1.4514 | 0.0000 |

**Table S4.** COI family proportion of sequences greater than 1%

| **Family** | **Primary** | **Old Secondary** | **Young Secondary** |
| --- | --- | --- | --- |
| Enchytraeidae | 33.1271 | 61.7891 | 36.2288 |
| Naididae | 19.3938 | 8.3140 | 11.5921 |
| Megascolecidae | 8.6060 | 4.2472 | 5.2660 |
| Isotomidae | 0.7443 | 0.5007 | 16.7991 |
| Elateridae | 12.3764 | 0.7656 | 0.0000 |
| Entomobryidae | 7.8777 | 2.6743 | 1.7974 |
| Glossoscolecidae | 2.5301 | 6.4460 | 2.2145 |
| Formicidae | 1.9513 | 3.5395 | 4.3565 |
| Onychiuridae | 2.1511 | 0.0000 | 6.8692 |
| Trhypochthoniidae | 2.0559 | 0.1897 | 6.2398 |
| Scolopocryptopidae | 0.0000 | 3.9542 | 0.0000 |
| Cicadellidae | 0.1082 | 2.7536 | 0.9429 |
| Termitidae | 0.6578 | 0.6143 | 2.3727 |
| Phoridae | 3.1400 | 0.0000 | 0.0000 |
| Chironomidae | 2.4277 | 0.1297 | 0.1196 |
| Lumbricidae | 0.1397 | 1.1137 | 0.7292 |
| Xerobdellidae | 0.0000 | 0.0000 | 1.6296 |
| Nitidulidae | 0.9868 | 0.0000 | 0.0000 |

**Table S5.** The Similarity of Percentage analysis (SIMPER) using a one-way design with a 70% cut-off percentage to list only higher-contributing taxonomic groups for the soil COI Class community composition between the three habitats in the MNWR (PF = primary forest, OS = 33-year-old secondary forest, YS = 23-year-old secondary forest).

| Pairwise Comparisons | Invertebrate Class | Average  Dissimilarity | Diss/SD | Percent  Contribution (%) | Cumulative  percent (%) |
| --- | --- | --- | --- | --- | --- |
| PF vs OS | Clitellata | 7.33 | 2.91 | 19.99 | 19.99 |
|  | Collembola | 4.12 | 0.87 | 11.25 | 31.23 |
|  | Insecta | 4.01 | 1.62 | 10.93 | 42.16 |
|  | Chilopoda | 3.33 | 1.63 | 9.09 | 51.25 |
|  | Arachnida | 3.01 | 1.38 | 8.21 | 59.47 |
|  | Polychaeta | 2.79 | 1.31 | 7.62 | 67.08 |
|  | Chromadorea | 2.72 | 1.57 | 7.41 | 74.50 |
| OS vs YS | Clitellata | 7.31 | 1.68 | 19.71 | 19.71 |
|  | Collembola | 5.75 | 0.87 | 15.51 | 35.22 |
|  | Arachnida | 3.99 | 1.38 | 10.77 | 45.99 |
|  | Chilopoda | 3.05 | 1.53 | 8.23 | 54.22 |
|  | Insecta | 2.89 | 1.10 | 7.79 | 62.01 |
|  | Polychaeta | 2.80 | 1.41 | 7.54 | 69.55 |
|  | Malacostraca | 1.86 | 1.15 | 5.03 | 74.58 |
| PF vs YS | Collembola | 6.04 | 1.03 | 16.69 | 16.69 |
|  | Clitellata | 5.05 | 1.02 | 13.96 | 30.65 |
|  | Insecta | 4.87 | 1.36 | 13.47 | 44.12 |
|  | Arachnida | 4.40 | 1.36 | 12.16 | 56.28 |
|  | Polychaeta | 2.97 | 1.18 | 8.21 | 64.50 |
|  | Chromadorea | 2.92 | 1.44 | 8.06 | 72.56 |

**Table S6.** The Similarity of Percentage analysis (SIMPER) using a one-way design with a 70% cut-off percentage to list only higher-contributing taxonomic groups for the soil COI Order community composition between the three habitats in the MNWR (PF = primary forest, OS = 33-year-old secondary forest, YS = 23-year-old secondary forest).

| Pairwise Comparisons | Invertebrate Order | Average  Dissimilarity | Diss/SD | Percent  Contribution (%) | Cumulative  percent (%) |
| --- | --- | --- | --- | --- | --- |
| PF vs OS | Haplotaxida | 4.77 | 2.85 | 9.31 | 9.31 |
|  | Entomobryomorpha | 2.56 | 0.84 | 4.99 | 14.30 |
|  | Coleoptera | 2.13 | 1.03 | 4.15 | 18.45 |
|  | Lepidoptera | 2.00 | 1.18 | 3.91 | 22.36 |
|  | Scolopendromorpha | 1.78 | 1.29 | 3.47 | 25.83 |
| OS vs YS | Haplotaxida | 5.16 | 1.61 | 9.81 | 9.81 |
|  | Entomobryomorpha | 4.04 | 0.83 | 7.67 | 17.49 |
|  | Sarcoptiformes | 3.02 | 1.50 | 5.74 | 23.23 |
|  | Lepidoptera | 2.79 | 1.47 | 5.30 | 28.52 |
|  | Phyllodocida | 1.98 | 1.18 | 3.76 | 32.28 |
| PF vs YS | Entomobryomorpha | 4.63 | 1.09 | 8.63 | 8.63 |
|  | Haplotaxida | 3.53 | 0.91 | 6.58 | 15.22 |
|  | Sarcoptiformes | 2.94 | 1.38 | 5.48 | 20.70 |
|  | Coleoptera | 2.64 | 1.08 | 4.93 | 25.63 |
|  | Blattodea | 2.32 | 0.88 | 4.34 | 29.97 |

**Table S7.** The Similarity of Percentage analysis (SIMPER) using a one-way design with a 70% cut-off percentage to list only higher-contributing taxonomic groups for the soil COI Family community composition between the three habitats in the MNWR (PF = primary forest, OS = 33-year-old secondary forest, YS = 23-year-old secondary forest)**.**

| Habitat  Pairwise Comparisons | Invertebrate Family | Average  Dissimilarity | Diss/SD | Percent  contribution (%) | Cumulative  percent (%) |
| --- | --- | --- | --- | --- | --- |
| PF vs OS | Enchytraeidae | 4.51 | 1.74 | 9.14 | 9.14 |
|  | Naididae | 4.37 | 1.05 | 8.85 | 17.99 |
|  | Elateridae | 3.91 | 0.60 | 7.93 | 25.92 |
|  | Glossoscolecidae | 3.59 | 1.64 | 7.27 | 33.19 |
|  | Megascolecidae | 3.57 | 1.44 | 7.23 | 40.42 |
|  | Entomobryidae | 2.93 | 0.97 | 5.93 | 46.35 |
| OS vs YS | Isotomidae | 5.27 | 0.59 | 10.41 | 10.41 |
|  | Enchytraeidae | 5.00 | 0.88 | 9.89 | 20.30 |
|  | Onychiuridae | 4.01 | 1.13 | 7.93 | 28.23 |
|  | Naididae | 3.80 | 1.52 | 7.52 | 35.74 |
|  | Trhypochthoniidae | 3.69 | 1.12 | 7.29 | 43.03 |
|  | Formicidae | 3.18 | 1.32 | 6.28 | 49.31 |
| PF vs YS | Naididae | 5.62 | 1.03 | 10.30 | 10.30 |
|  | Isotomidae | 5.12 | 0.60 | 9.40 | 19.70 |
|  | Enchytraeidae | 3.94 | 1.06 | 7.22 | 26.92 |
|  | Trhypochthoniidae | 3.82 | 1.10 | 7.01 | 33.93 |
|  | Onychiuridae | 3.68 | 1.20 | 6.75 | 40.68 |
|  | Elateridae | 3.60 | 0.52 | 6.60 | 47.28 |
|  | Megascolecidae | 3.51 | 1.31 | 6.44 | 53.72 |
|  | Entomobryidae | 3.43 | 0.96 | 6.30 | 60.02 |

|  |  | **Primary** | **Old Secondary** | **Young Secondary** |
| --- | --- | --- | --- | --- |
| **(a) Class** | Clitellata | 22.4988 | 48.9432 | 14.5616 |
|  | Insecta | 30.6732 | 24.9377 | 21.0466 |
|  | Collembola | 19.1567 | 3.2123 | 28.3412 |
|  | Arachnida | 10.8713 | 5.5153 | 17.8634 |
|  | Chromadorea | 9.8300 | 4.1655 | 5.3960 |
|  | Polychaeta | 0.6159 | 5.0755 | 5.9399 |
|  | Chilopoda | 0.8365 | 4.6633 | 0.5128 |
|  | Malacostraca | 1.7944 | 1.2544 | 1.8981 |
|  | Gastropoda | 1.6638 | 0.7189 | 2.2374 |
|  | Cestoda | 1.4566 | 0.2197 | 1.2846 |
| **(b) Order** |  |  |  |  |
|  | Haplotaxida | 22.4470 | 48.8905 | 14.2038 |
|  | Entomobryomorpha | 17.2021 | 3.0921 | 26.4398 |
|  | Coleoptera | 11.3763 | 2.8190 | 1.7546 |
|  | Ascaridida | 7.8369 | 3.1835 | 4.5340 |
|  | Sarcoptiformes | 0.6236 | 1.0886 | 11.3084 |
|  | Hemiptera | 1.5697 | 3.4324 | 6.2703 |
|  | Lepidoptera | 0.8123 | 6.7572 | 3.3075 |
|  | Araneae | 3.2667 | 2.9601 | 4.5972 |
|  | Blattodea | 7.7094 | 0.3793 | 2.2808 |
|  | Odonata | 3.2403 | 2.7499 | 4.3690 |
|  | Phyllodocida | 0.4716 | 2.9642 | 4.2590 |
|  | Diptera | 2.4211 | 3.1152 | 0.8018 |
|  | Ixodida | 4.3879 | 0.1475 | 0.0782 |
|  | Hymenoptera | 0.6250 | 2.2938 | 1.4912 |
|  | Decapoda | 1.4229 | 1.0588 | 1.7807 |
|  | Scorpiones | 2.2992 | 0.7171 | 1.1025 |
|  | Poduromorpha | 1.9529 | 0.1202 | 1.9014 |
|  | Scolopendromorpha | 0.0764 | 3.2119 | 0.5128 |
|  | Stylommatophora | 1.5036 | 0.4918 | 1.6980 |
|  | undef_Polychaeta | 0.0000 | 1.3571 | 1.4576 |
|  | Ephemeroptera | 1.8422 | 0.3384 | 0.0000 |
|  | Phasmatodea | 0.5487 | 0.8312 | 0.4099 |
|  | Rhabditida | 1.0629 | 0.1936 | 0.3317 |
|  | Orthoptera | 0.0674 | 1.4402 | 0.0000 |
|  | Geophilomorpha | 0.0000 | 1.4514 | 0.0000 |
| **(c) Family** |  |  |  |  |
|  | Enchytraeidae | 33.1271 | 61.7891 | 36.2288 |
|  | Naididae | 19.3938 | 8.3140 | 11.5921 |
|  | Megascolecidae | 8.6060 | 4.2472 | 5.2660 |
|  | Isotomidae | 0.7443 | 0.5007 | 16.7991 |
|  | Elateridae | 12.3764 | 0.7656 | 0.0000 |
|  | Entomobryidae | 7.8777 | 2.6743 | 1.7974 |
|  | Glossoscolecidae | 2.5301 | 6.4460 | 2.2145 |
|  | Formicidae | 1.9513 | 3.5395 | 4.3565 |
|  | Onychiuridae | 2.1511 | 0.0000 | 6.8692 |
|  | Trhypochthoniidae | 2.0559 | 0.1897 | 6.2398 |
|  | Scolopocryptopidae | 0.0000 | 3.9542 | 0.0000 |
|  | Cicadellidae | 0.1082 | 2.7536 | 0.9429 |
|  | Termitidae | 0.6578 | 0.6143 | 2.3727 |
|  | Phoridae | 3.1400 | 0.0000 | 0.0000 |
|  | Chironomidae | 2.4277 | 0.1297 | 0.1196 |
|  | Lumbricidae | 0.1397 | 1.1137 | 0.7292 |
|  | Xerobdellidae | 0.0000 | 0.0000 | 1.6296 |
|  | Nitidulidae | 0.9868 | 0.0000 | 0.0000 |
